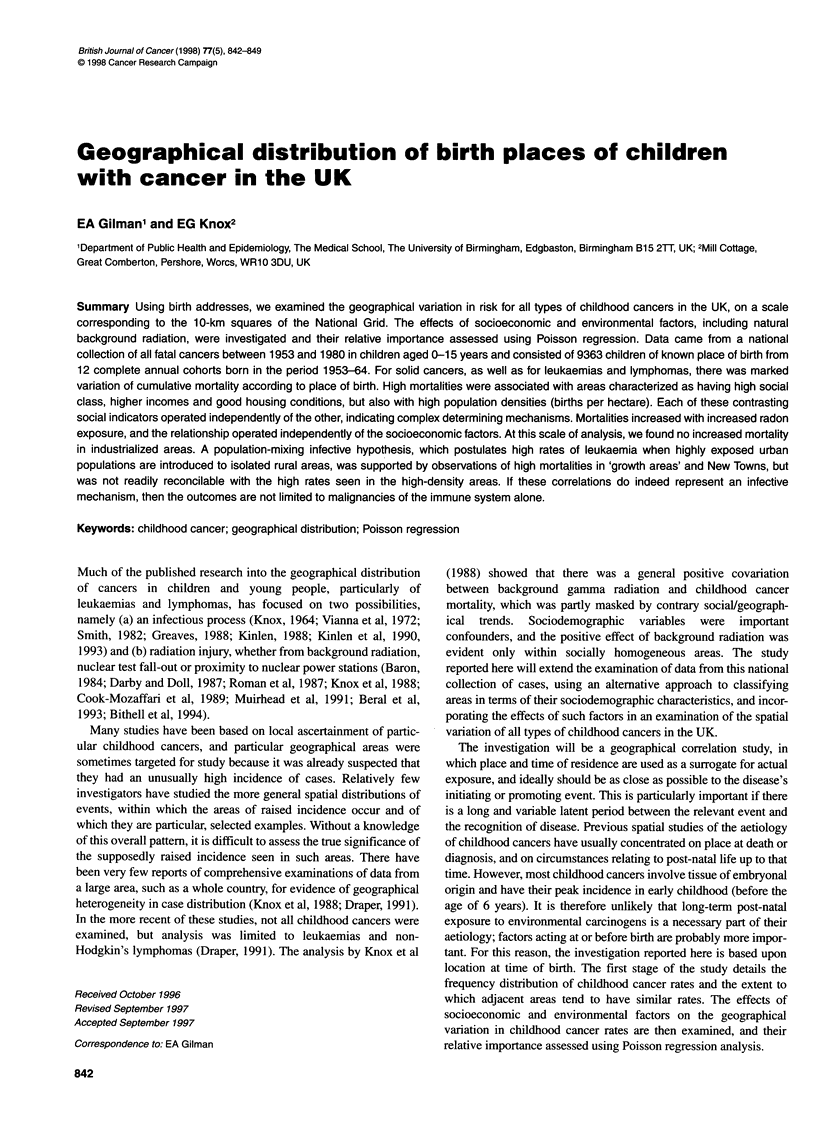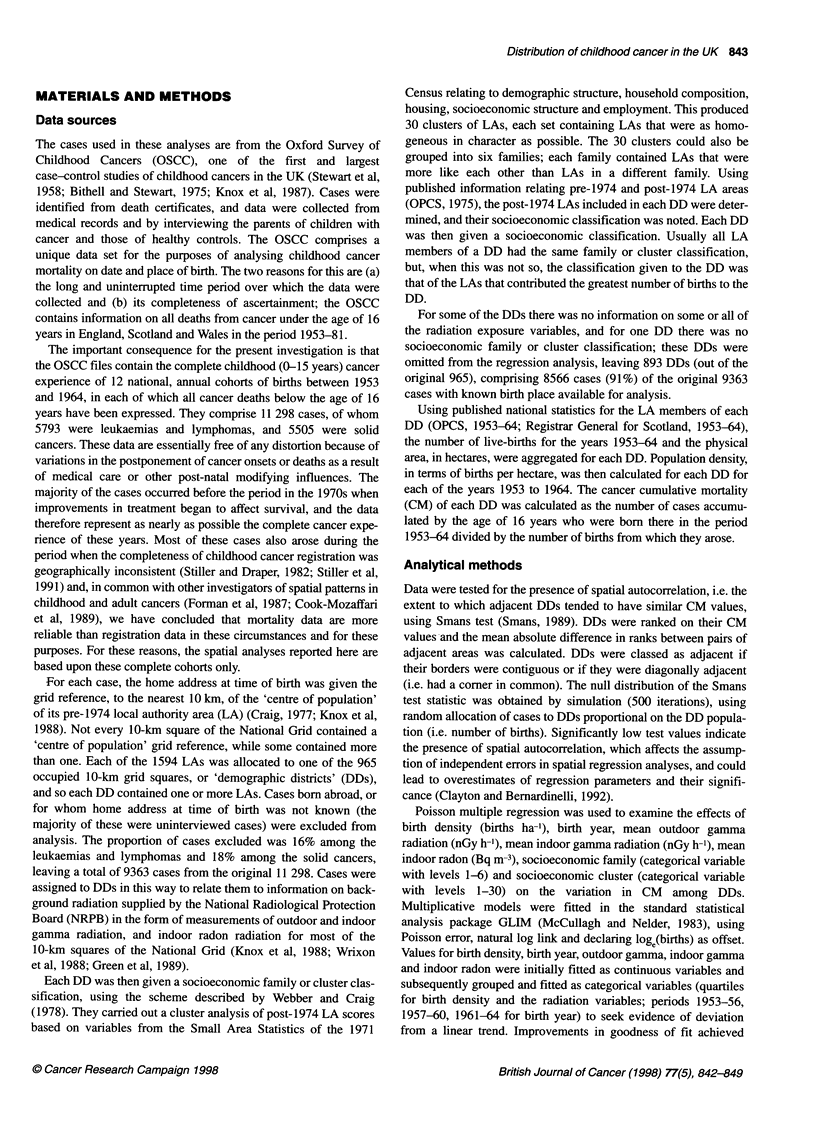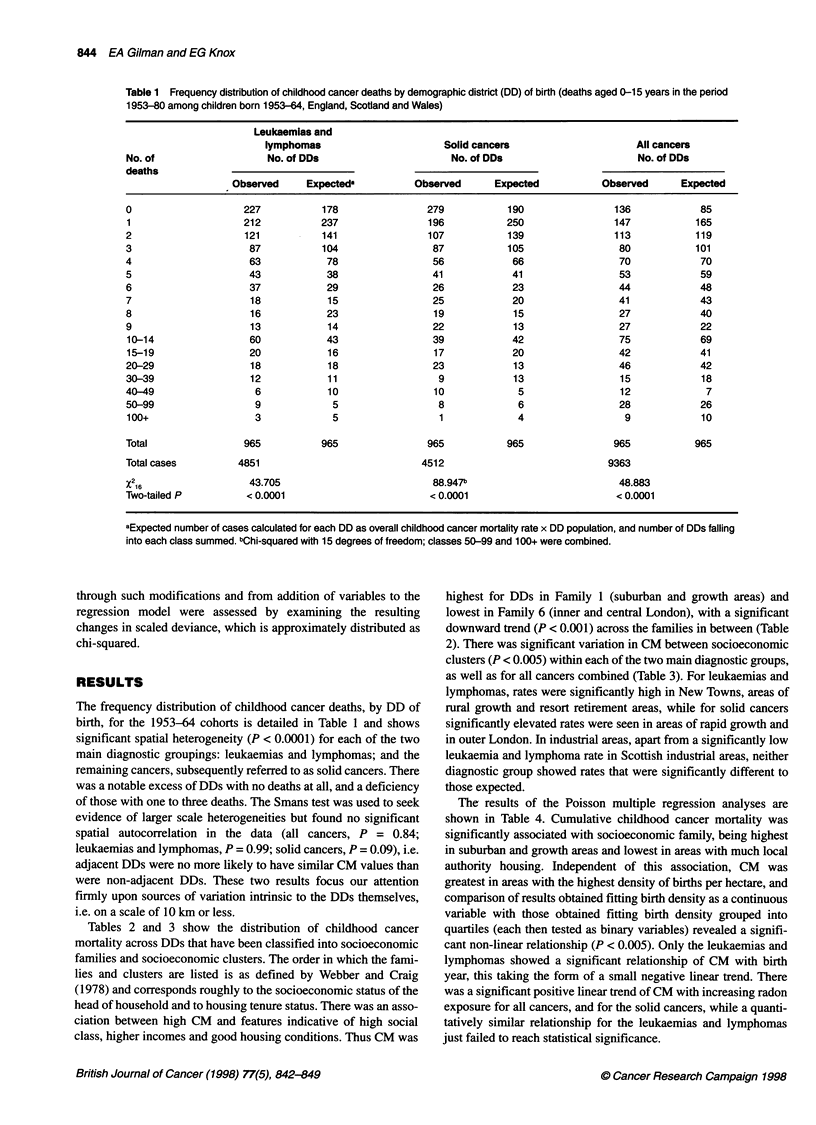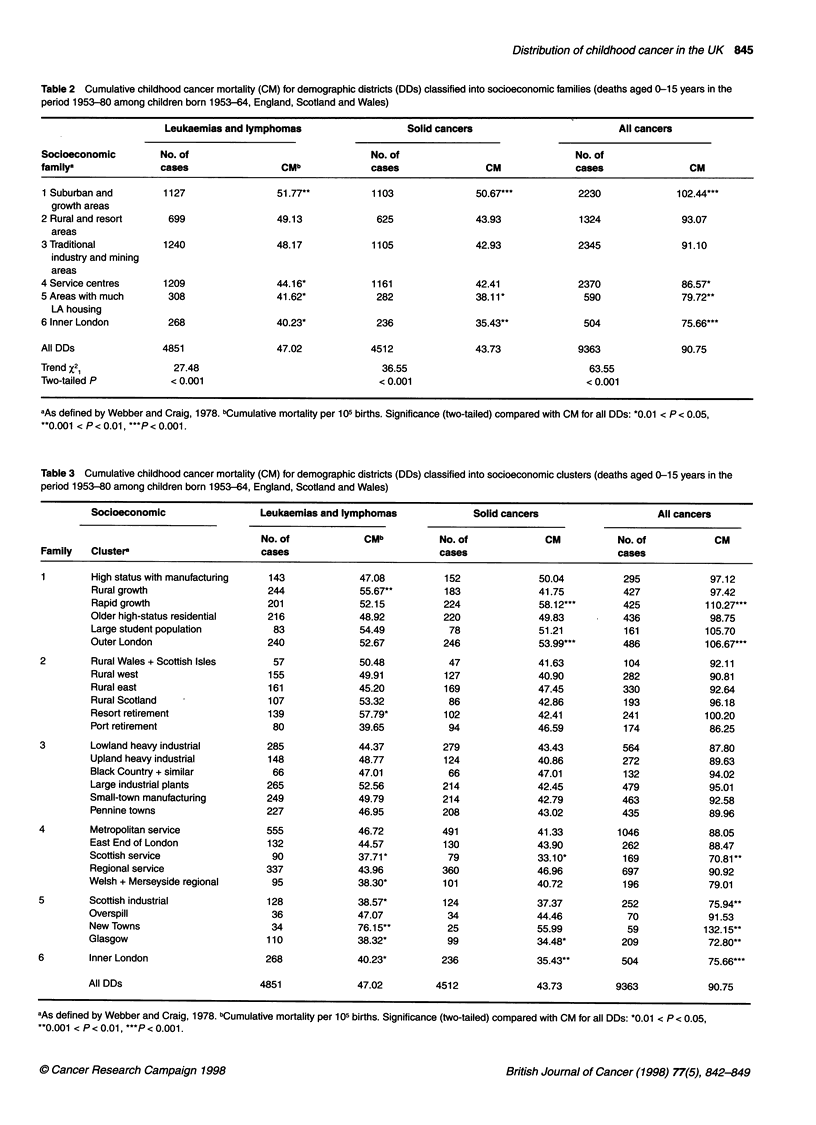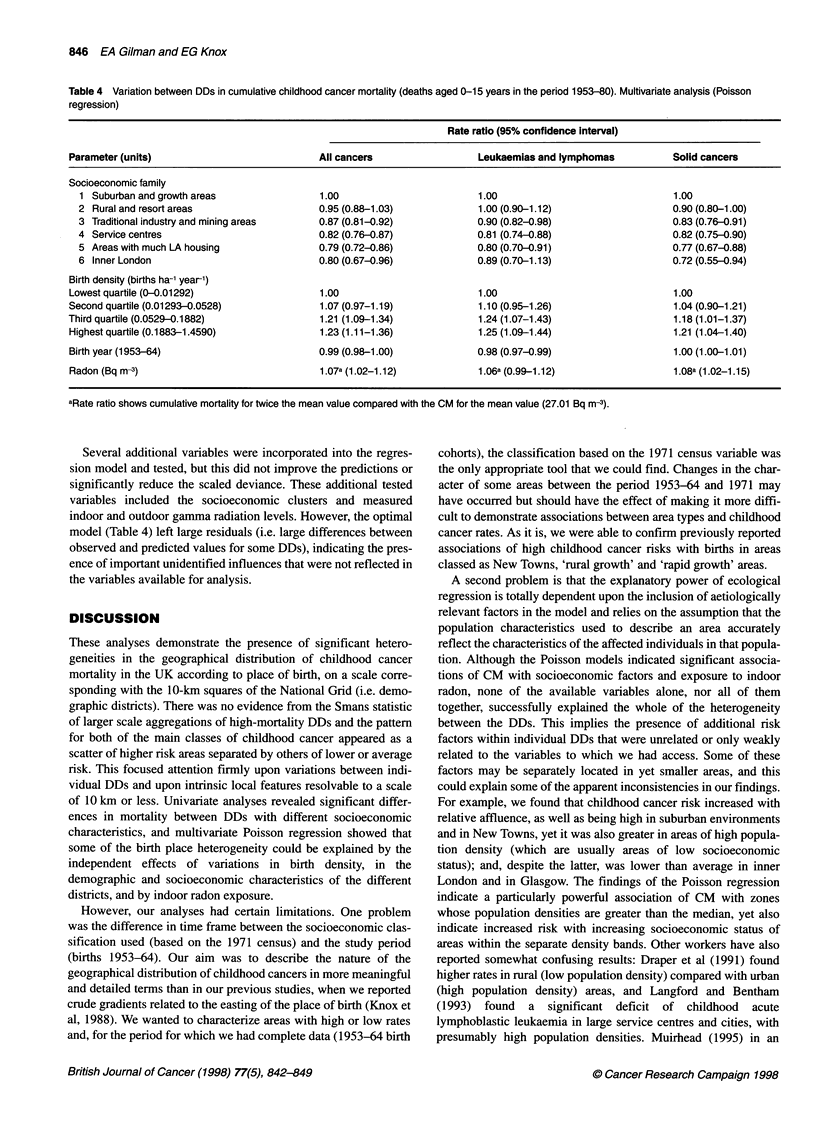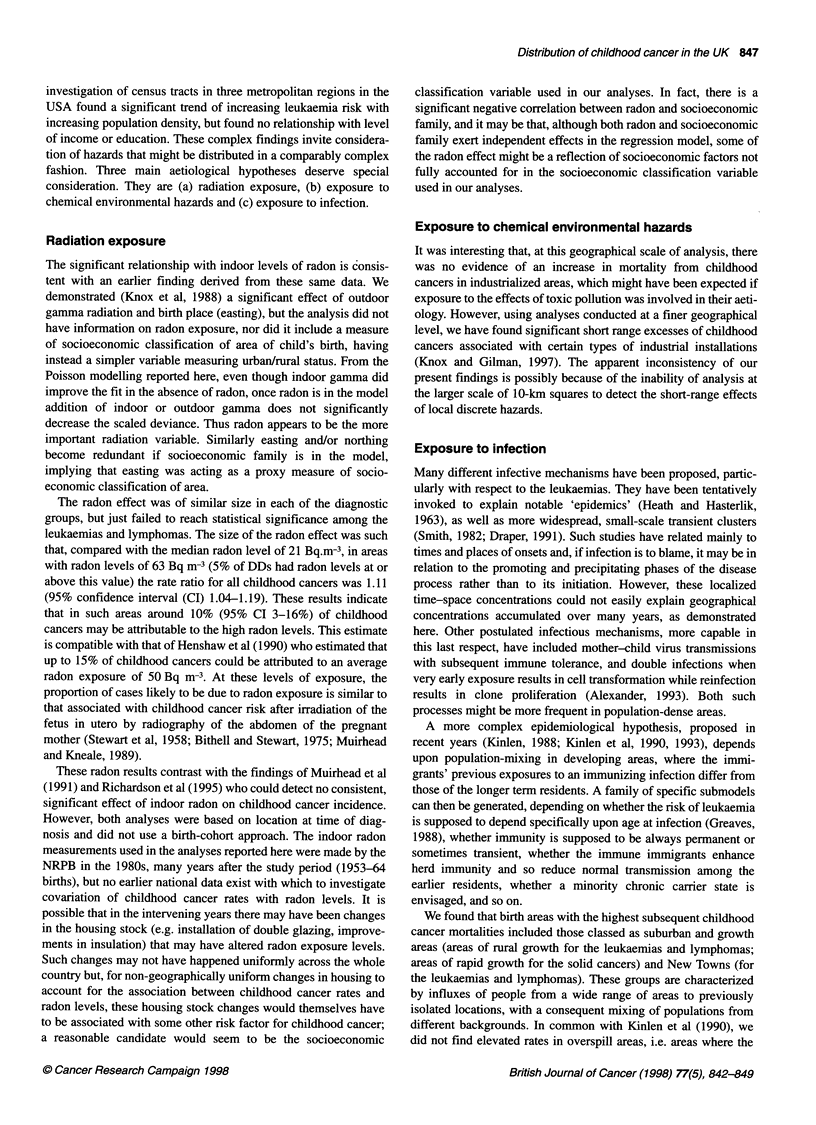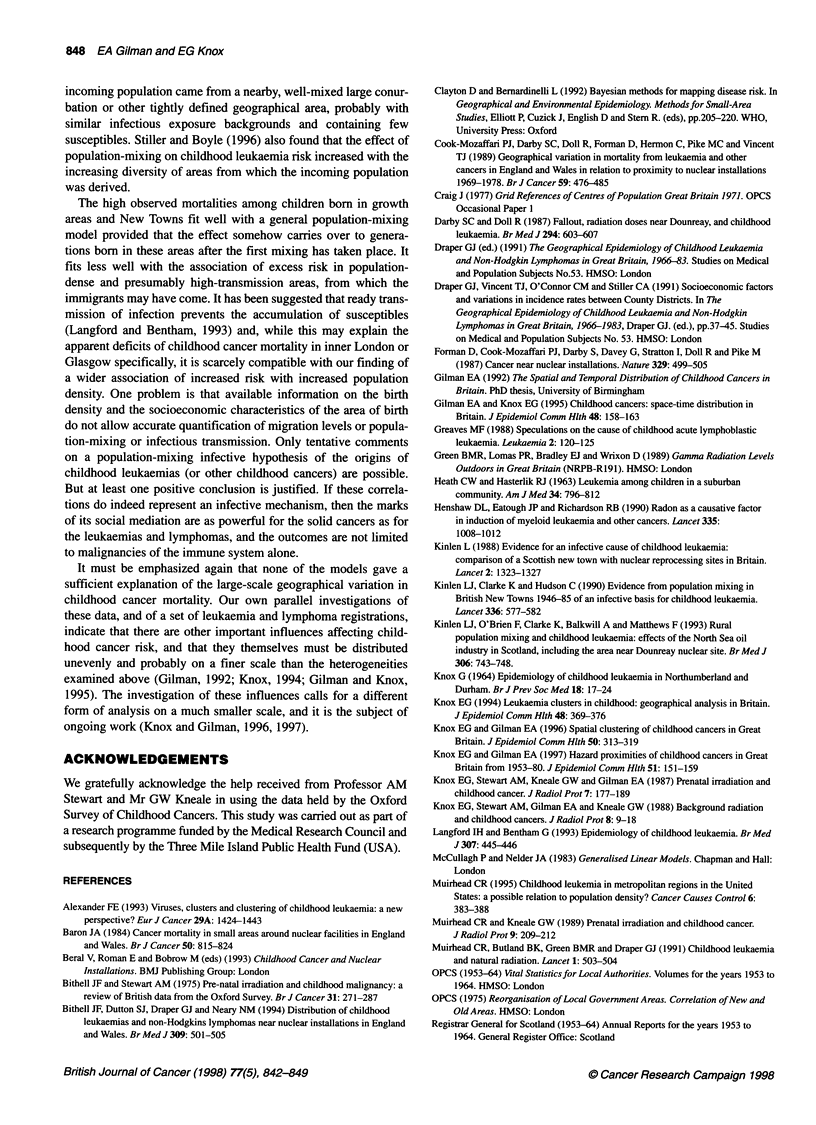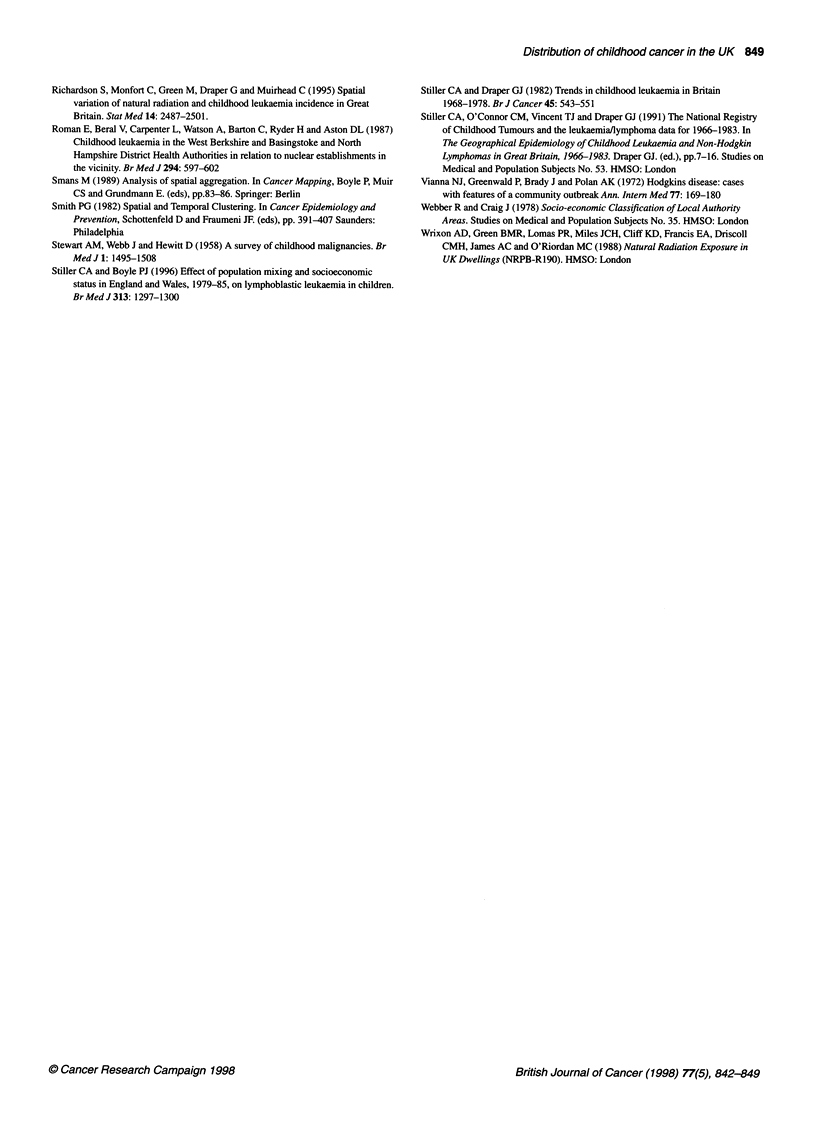# Geographical distribution of birth places of children with cancer in the UK.

**DOI:** 10.1038/bjc.1998.137

**Published:** 1998-03

**Authors:** E. A. Gilman, E. G. Knox

**Affiliations:** Department of Public Health and Epidemiology, The Medical School, The University of Birmingham, Edgbaston, UK.

## Abstract

Using birth addresses, we examined the geographical variation in risk for all types of childhood cancers in the UK, on a scale corresponding to the 10-km squares of the National Grid. The effects of socioeconomic and environmental factors, including natural background radiation, were investigated and their relative importance assessed using Poisson regression. Data came from a national collection of all fatal cancers between 1953 and 1980 in children aged 0-15 years and consisted of 9363 children of known place of birth from 12 complete annual cohorts born in the period 1953-64. For solid cancers, as well as for leukaemias and lymphomas, there was marked variation of cumulative mortality according to place of birth. High mortalities were associated with areas characterized as having high social class, higher incomes and good housing conditions, but also with high population densities (births per hectare). Each of these contrasting social indicators operated independently of the other, indicating complex determining mechanisms. Mortalities increased with increased radon exposure, and the relationship operated independently of the socioeconomic factors. At this scale of analysis, we found no increased mortality in industrialized areas. A population-mixing infective hypothesis, which postulates high rates of leukaemia when highly exposed urban populations are introduced to isolated rural areas, was supported by observations of high mortalities in 'growth areas' and New Towns, but was not readily reconcilable with the high rates seen in the high-density areas. If these correlations do indeed represent an infective mechanism, then the outcomes are not limited to malignancies of the immune system alone.


British Journal of Cancer (1998) 77(5), 842–849
© 1998 Cancer Research Campaign

### Geographical distribution of birth places of children with cancer in the UK

**EA Gilman[1] and EG Knox[2]**

[1]Department of Public Health and Epidemiology, The Medical School, The University of Birmingham, Edgbaston, Birmingham B15 2TT, UK; [2]Mill Cottage, Great Comberton, Pershore, Worcs, WR10 3DU, UK

**Summary** Using birth addresses, we examined the geographical variation in risk for all types of childhood cancers in the UK, on a scale corresponding to the 10-km squares of the National Grid. The effects of socioeconomic and environmental factors, including natural background radiation, were investigated and their relative importance assessed using Poisson regression. Data came from a national collection of all fatal cancers between 1953 and 1980 in children aged 0–15 years and consisted of 9363 children of known place of birth from 12 complete annual cohorts born in the period 1953–64. For solid cancers, as well as for leukaemias and lymphomas, there was marked variation of cumulative mortality according to place of birth. High mortalities were associated with areas characterized as having high social class, higher incomes and good housing conditions, but also with high population densities (births per hectare). Each of these contrasting social indicators operated independently of the other, indicating complex determining mechanisms. Mortalities increased with increased radon exposure, and the relationship operated independently of the socioeconomic factors. At this scale of analysis, we found no increased mortality in industrialized areas. A population-mixing infective hypothesis, which postulates high rates of leukaemia when highly exposed urban populations are introduced to isolated rural areas, was supported by observations of high mortalities in 'growth areas' and New Towns, but was not readily reconcilable with the high rates seen in the high-density areas. If these correlations do indeed represent an infective mechanism, then the outcomes are not limited to malignancies of the immune system alone.

**Keywords:** childhood cancer; geographical distribution; Poisson regression

Much of the published research into the geographical distribution of cancers in children and young people, particularly of leukaemias and lymphomas, has focused on two possibilities, namely (a) an infectious process (Knox, 1964; Vianna et al, 1972; Smith, 1982; Greaves, 1988; Kinlen, 1988; Kinlen et al, 1990, 1993) and (b) radiation injury, whether from background radiation, nuclear test fall-out or proximity to nuclear power stations (Baron, 1984; Darby and Doll, 1987; Roman et al, 1987; Knox et al, 1988; Cook-Mozaffari et al, 1989; Muirhead et al, 1991; Beral et al, 1993; Bithell et al, 1994).

Many studies have been based on local ascertainment of particular childhood cancers, and particular geographical areas were sometimes targeted for study because it was already suspected that they had an unusually high incidence of cases. Relatively few investigators have studied the more general spatial distributions of events, within which the areas of raised incidence occur and of which they are particular, selected examples. Without a knowledge of this overall pattern, it is difficult to assess the true significance of the supposedly raised incidence seen in such areas. There have been very few reports of comprehensive examinations of data from a large area, such as a whole country, for evidence of geographical heterogeneity in case distribution (Knox et al, 1988; Draper, 1991). In the more recent of these studies, not all childhood cancers were examined, but analysis was limited to leukaemias and non-Hodgkin's lymphomas (Draper, 1991). The analysis by Knox et al (1988) showed that there was a general positive covariation between background gamma radiation and childhood cancer mortality, which was partly masked by contrary social/geographical trends. Sociodemographic variables were important confounders, and the positive effect of background radiation was evident only within socially homogeneous areas. The study reported here will extend the examination of data from this national collection of cases, using an alternative approach to classifying areas in terms of their sociodemographic characteristics, and incorporating the effects of such factors in an examination of the spatial variation of all types of childhood cancers in the UK.

The investigation will be a geographical correlation study, in which place and time of residence are used as a surrogate for actual exposure, and ideally should be as close as possible to the disease's initiating or promoting event. This is particularly important if there is a long and variable latent period between the relevant event and the recognition of disease. Previous spatial studies of the aetiology of childhood cancers have usually concentrated on place at death or diagnosis, and on circumstances relating to post-natal life up to that time. However, most childhood cancers involve tissue of embryonal origin and have their peak incidence in early childhood (before the age of 6 years). It is therefore unlikely that long-term post-natal exposure to environmental carcinogens is a necessary part of their aetiology; factors acting at or before birth are probably more important. For this reason, the investigation reported here is based upon location at time of birth. The first stage of the study details the frequency distribution of childhood cancer rates and the extent to which adjacent areas tend to have similar rates. The effects of socioeconomic and environmental factors on the geographical variation in childhood cancer rates are then examined, and their relative importance assessed using Poisson regression analysis.

*Received October 1996*
*Revised September 1997*
*Accepted September 1997*

*Correspondence to:* EA Gilman

### MATERIALS AND METHODS

### Data sources

The cases used in these analyses are from the Oxford Survey of Childhood Cancers (OSCC), one of the first and largest case–control studies of childhood cancers in the UK (Stewart et al, 1958; Bithell and Stewart, 1975; Knox et al, 1987). Cases were identified from death certificates, and data were collected from medical records and by interviewing the parents of children with cancer and those of healthy controls. The OSCC comprises a unique data set for the purposes of analysing childhood cancer mortality on date and place of birth. The two reasons for this are (a) the long and uninterrupted time period over which the data were collected and (b) its completeness of ascertainment; the OSCC contains information on all deaths from cancer under the age of 16 years in England, Scotland and Wales in the period 1953–81.

The important consequence for the present investigation is that the OSCC files contain the complete childhood (0–15 years) cancer experience of 12 national, annual cohorts of births between 1953 and 1964, in each of which all cancer deaths below the age of 16 years have been expressed. They comprise 11 298 cases, of whom 5793 were leukaemias and lymphomas, and 5505 were solid cancers. These data are essentially free of any distortion because of variations in the postponement of cancer onsets or deaths as a result of medical care or other post-natal modifying influences. The majority of the cases occurred before the period in the 1970s when improvements in treatment began to affect survival, and the data therefore represent as nearly as possible the complete cancer experience of these years. Most of these cases also arose during the period when the completeness of childhood cancer registration was geographically inconsistent (Stiller and Draper, 1982; Stiller et al, 1991) and, in common with other investigators of spatial patterns in childhood and adult cancers (Forman et al, 1987; Cook-Mozaffari et al, 1989), we have concluded that mortality data are more reliable than registration data in these circumstances and for these purposes. For these reasons, the spatial analyses reported here are based upon these complete cohorts only.

For each case, the home address at time of birth was given the grid reference, to the nearest 10 km, of the 'centre of population' of its pre-1974 local authority area (LA) (Craig, 1977; Knox et al, 1988). Not every 10-km square of the National Grid contained a 'centre of population' grid reference, while some contained more than one. Each of the 1594 LAs was allocated to one of the 965 occupied 10-km grid squares, or 'demographic districts' (DDs), and so each DD contained one or more LAs. Cases born abroad, or for whom home address at time of birth was not known (the majority of these were uninterviewed cases) were excluded from analysis. The proportion of cases excluded was 16% among the leukaemias and lymphomas and 18% among the solid cancers, leaving a total of 9363 cases from the original 11 298. Cases were assigned to DDs in this way to relate them to information on background radiation supplied by the National Radiological Protection Board (NRPB) in the form of measurements of outdoor and indoor gamma radiation, and indoor radon radiation for most of the 10-km squares of the National Grid (Knox et al, 1988; Wrixon et al, 1988; Green et al, 1989).

Each DD was then given a socioeconomic family or cluster classification, using the scheme described by Webber and Craig (1978). They carried out a cluster analysis of post-1974 LA scores based on variables from the Small Area Statistics of the 1971 Census relating to demographic structure, household composition, housing, socioeconomic structure and employment. This produced 30 clusters of LAs, each set containing LAs that were as homogeneous in character as possible. The 30 clusters could also be grouped into six families; each family contained LAs that were more like each other than LAs in a different family. Using published information relating pre-1974 and post-1974 LA areas (OPCS, 1975), the post-1974 LAs included in each DD were determined, and their socioeconomic classification was noted. Each DD was then given a socioeconomic classification. Usually all LA members of a DD had the same family or cluster classification, but, when this was not so, the classification given to the DD was that of the LAs that contributed the greatest number of births to the DD.

For some of the DDs there was no information on some or all of the radiation exposure variables, and for one DD there was no socioeconomic family or cluster classification; these DDs were omitted from the regression analysis, leaving 893 DDs (out of the original 965), comprising 8566 cases (91%) of the original 9363 cases with known birth place available for analysis.

Using published national statistics for the LA members of each DD (OPCS, 1953–64; Registrar General for Scotland, 1953–64), the number of live-births for the years 1953–64 and the physical area, in hectares, were aggregated for each DD. Population density, in terms of births per hectare, was then calculated for each DD for each of the years 1953 to 1964. The cancer cumulative mortality (CM) of each DD was calculated as the number of cases accumulated by the age of 16 years who were born there in the period 1953–64 divided by the number of births from which they arose.

### Analytical methods

Data were tested for the presence of spatial autocorrelation, i.e. the extent to which adjacent DDs tended to have similar CM values, using Smans test (Smans, 1989). DDs were ranked on their CM values and the mean absolute difference in ranks between pairs of adjacent areas was calculated. DDs were classed as adjacent if their borders were contiguous or if they were diagonally adjacent (i.e. had a corner in common). The null distribution of the Smans test statistic was obtained by simulation (500 iterations), using random allocation of cases to DDs proportional on the DD population (i.e. number of births). Significantly low test values indicate the presence of spatial autocorrelation, which affects the assumption of independent errors in spatial regression analyses, and could lead to overestimates of regression parameters and their significance (Clayton and Bernardinelli, 1992).

Poisson multiple regression was used to examine the effects of birth density (births $ha^{-1}$), birth year, mean outdoor gamma radiation (nGy $h^{-1}$), mean indoor gamma radiation (nGy $h^{-1}$), mean indoor radon (Bq $m^{-3}$), socioeconomic family (categorical variable with levels 1–6) and socioeconomic cluster (categorical variable with levels 1–30) on the variation in CM among DDs. Multiplicative models were fitted in the standard statistical analysis package GLIM (McCullagh and Nelder, 1983), using Poisson error, natural log link and declaring $\log_e$(births) as offset. Values for birth density, birth year, outdoor gamma, indoor gamma and indoor radon were initially fitted as continuous variables and subsequently grouped and fitted as categorical variables (quartiles for birth density and the radiation variables; periods 1953–56, 1957–60, 1961–64 for birth year) to seek evidence of deviation from a linear trend. Improvements in goodness of fit achieved

© Cancer Research Campaign 1998

**Table 1** Frequency distribution of childhood cancer deaths by demographic district (DD) of birth (deaths aged 0–15 years in the period 1953–80 among children born 1953–64, England, Scotland and Wales)

| No. of deaths | Leukaemias and lymphomas No. of DDs | | Solid cancers No. of DDs | | All cancers No. of DDs | |
|---|---|---|---|---|---|---|
| | Observed | Expected[a] | Observed | Expected | Observed | Expected |
| 0 | 227 | 178 | 279 | 190 | 136 | 85 |
| 1 | 212 | 237 | 196 | 250 | 147 | 165 |
| 2 | 121 | 141 | 107 | 139 | 113 | 119 |
| 3 | 87 | 104 | 87 | 105 | 80 | 101 |
| 4 | 63 | 78 | 56 | 66 | 70 | 70 |
| 5 | 43 | 38 | 41 | 41 | 53 | 59 |
| 6 | 37 | 29 | 26 | 23 | 44 | 48 |
| 7 | 18 | 15 | 25 | 20 | 41 | 43 |
| 8 | 16 | 23 | 19 | 15 | 27 | 40 |
| 9 | 13 | 14 | 22 | 13 | 27 | 22 |
| 10–14 | 60 | 43 | 39 | 42 | 75 | 69 |
| 15–19 | 20 | 16 | 17 | 20 | 42 | 41 |
| 20–29 | 18 | 18 | 23 | 13 | 46 | 42 |
| 30–39 | 12 | 11 | 9 | 13 | 15 | 18 |
| 40–49 | 6 | 10 | 10 | 5 | 12 | 7 |
| 50–99 | 9 | 5 | 8 | 6 | 28 | 26 |
| 100+ | 3 | 5 | 1 | 4 | 9 | 10 |
| Total | 965 | 965 | 965 | 965 | 965 | 965 |
| Total cases | 4851 | | 4512 | | 9363 | |
| $\chi^2_{16}$ | 43.705 | | 88.947[b] | | 48.883 | |
| Two-tailed *P* | < 0.0001 | | < 0.0001 | | < 0.0001 | |

[a]Expected number of cases calculated for each DD as overall childhood cancer mortality rate × DD population, and number of DDs falling into each class summed. [b]Chi-squared with 15 degrees of freedom; classes 50–99 and 100+ were combined.

through such modifications and from addition of variables to the regression model were assessed by examining the resulting changes in scaled deviance, which is approximately distributed as chi-squared.

### RESULTS

The frequency distribution of childhood cancer deaths, by DD of birth, for the 1953–64 cohorts is detailed in Table 1 and shows significant spatial heterogeneity ($P < 0.0001$) for each of the two main diagnostic groupings: leukaemias and lymphomas; and the remaining cancers, subsequently referred to as solid cancers. There was a notable excess of DDs with no deaths at all, and a deficiency of those with one to three deaths. The Smans test was used to seek evidence of larger scale heterogeneities but found no significant spatial autocorrelation in the data (all cancers, $P = 0.84$; leukaemias and lymphomas, $P = 0.99$; solid cancers, $P = 0.09$), i.e. adjacent DDs were no more likely to have similar CM values than were non-adjacent DDs. These two results focus our attention firmly upon sources of variation intrinsic to the DDs themselves, i.e. on a scale of 10 km or less.

Tables 2 and 3 show the distribution of childhood cancer mortality across DDs that have been classified into socioeconomic families and socioeconomic clusters. The order in which the families and clusters are listed is as defined by Webber and Craig (1978) and corresponds roughly to the socioeconomic status of the head of household and to housing tenure status. There was an association between high CM and features indicative of high social class, higher incomes and good housing conditions. Thus CM was highest for DDs in Family 1 (suburban and growth areas) and lowest in Family 6 (inner and central London), with a significant downward trend ($P < 0.001$) across the families in between (Table 2). There was significant variation in CM between socioeconomic clusters ($P < 0.005$) within each of the two main diagnostic groups, as well as for all cancers combined (Table 3). For leukaemias and lymphomas, rates were significantly high in New Towns, areas of rural growth and resort retirement areas, while for solid cancers significantly elevated rates were seen in areas of rapid growth and in outer London. In industrial areas, apart from a significantly low leukaemia and lymphoma rate in Scottish industrial areas, neither diagnostic group showed rates that were significantly different to those expected.

The results of the Poisson multiple regression analyses are shown in Table 4. Cumulative childhood cancer mortality was significantly associated with socioeconomic family, being highest in suburban and growth areas and lowest in areas with much local authority housing. Independent of this association, CM was greatest in areas with the highest density of births per hectare, and comparison of results obtained fitting birth density as a continuous variable with those obtained fitting birth density grouped into quartiles (each then tested as binary variables) revealed a significant non-linear relationship ($P < 0.005$). Only the leukaemias and lymphomas showed a significant relationship of CM with birth year, this taking the form of a small negative linear trend. There was a significant positive linear trend of CM with increasing radon exposure for all cancers, and for the solid cancers, while a quantitatively similar relationship for the leukaemias and lymphomas just failed to reach statistical significance.

 © Cancer Research Campaign 1998

**Table 2** Cumulative childhood cancer mortality (CM) for demographic districts (DDs) classified into socioeconomic families (deaths aged 0–15 years in the period 1953–80 among children born 1953–64, England, Scotland and Wales)

| | Leukaemias and lymphomas | | Solid cancers | | All cancers | |
|---|---|---|---|---|---|---|
| **Socioeconomic family**[a] | **No. of cases** | **CM**[b] | **No. of cases** | **CM** | **No. of cases** | **CM** |
| 1 Suburban and growth areas | 1127 | 51.77** | 1103 | 50.67*** | 2230 | 102.44*** |
| 2 Rural and resort areas | 699 | 49.13 | 625 | 43.93 | 1324 | 93.07 |
| 3 Traditional industry and mining areas | 1240 | 48.17 | 1105 | 42.93 | 2345 | 91.10 |
| 4 Service centres | 1209 | 44.16* | 1161 | 42.41 | 2370 | 86.57* |
| 5 Areas with much LA housing | 308 | 41.62* | 282 | 38.11* | 590 | 79.72** |
| 6 Inner London | 268 | 40.23* | 236 | 35.43** | 504 | 75.66*** |
| All DDs | 4851 | 47.02 | 4512 | 43.73 | 9363 | 90.75 |
| Trend $\chi^2_1$ | 27.48 | | 36.55 | | 63.55 | |
| Two-tailed $P$ | < 0.001 | | < 0.001 | | < 0.001 | |

[a]As defined by Webber and Craig, 1978. [b]Cumulative mortality per $10^5$ births. Significance (two-tailed) compared with CM for all DDs: *0.01 < $P$ < 0.05, **0.001 < $P$ < 0.01, ***$P$ < 0.001.

**Table 3** Cumulative childhood cancer mortality (CM) for demographic districts (DDs) classified into socioeconomic clusters (deaths aged 0–15 years in the period 1953–80 among children born 1953–64, England, Scotland and Wales)

| Socioeconomic | | Leukaemias and lymphomas | | Solid cancers | | All cancers | |
|---|---|---|---|---|---|---|---|
| **Family** | **Cluster**[a] | **No. of cases** | **CM**[b] | **No. of cases** | **CM** | **No. of cases** | **CM** |
| 1 | High status with manufacturing | 143 | 47.08 | 152 | 50.04 | 295 | 97.12 |
| | Rural growth | 244 | 55.67** | 183 | 41.75 | 427 | 97.42 |
| | Rapid growth | 201 | 52.15 | 224 | 58.12*** | 425 | 110.27*** |
| | Older high-status residential | 216 | 48.92 | 220 | 49.83 | 436 | 98.75 |
| | Large student population | 83 | 54.49 | 78 | 51.21 | 161 | 105.70 |
| | Outer London | 240 | 52.67 | 246 | 53.99*** | 486 | 106.67*** |
| 2 | Rural Wales + Scottish Isles | 57 | 50.48 | 47 | 41.63 | 104 | 92.11 |
| | Rural west | 155 | 49.91 | 127 | 40.90 | 282 | 90.81 |
| | Rural east | 161 | 45.20 | 169 | 47.45 | 330 | 92.64 |
| | Rural Scotland | 107 | 53.32 | 86 | 42.86 | 193 | 96.18 |
| | Resort retirement | 139 | 57.79* | 102 | 42.41 | 241 | 100.20 |
| | Port retirement | 80 | 39.65 | 94 | 46.59 | 174 | 86.25 |
| 3 | Lowland heavy industrial | 285 | 44.37 | 279 | 43.43 | 564 | 87.80 |
| | Upland heavy industrial | 148 | 48.77 | 124 | 40.86 | 272 | 89.63 |
| | Black Country + similar | 66 | 47.01 | 66 | 47.01 | 132 | 94.02 |
| | Large industrial plants | 265 | 52.56 | 214 | 42.45 | 479 | 95.01 |
| | Small-town manufacturing | 249 | 49.79 | 214 | 42.79 | 463 | 92.58 |
| | Pennine towns | 227 | 46.95 | 208 | 43.02 | 435 | 89.96 |
| 4 | Metropolitan service | 555 | 46.72 | 491 | 41.33 | 1046 | 88.05 |
| | East End of London | 132 | 44.57 | 130 | 43.90 | 262 | 88.47 |
| | Scottish service | 90 | 37.71* | 79 | 33.10* | 169 | 70.81** |
| | Regional service | 337 | 43.96 | 360 | 46.96 | 697 | 90.92 |
| | Welsh + Merseyside regional | 95 | 38.30* | 101 | 40.72 | 196 | 79.01 |
| 5 | Scottish industrial | 128 | 38.57* | 124 | 37.37 | 252 | 75.94** |
| | Overspill | 36 | 47.07 | 34 | 44.46 | 70 | 91.53 |
| | New Towns | 34 | 76.15** | 25 | 55.99 | 59 | 132.15** |
| | Glasgow | 110 | 38.32* | 99 | 34.48* | 209 | 72.80** |
| 6 | Inner London | 268 | 40.23* | 236 | 35.43** | 504 | 75.66*** |
| | All DDs | 4851 | 47.02 | 4512 | 43.73 | 9363 | 90.75 |

[a]As defined by Webber and Craig, 1978. [b]Cumulative mortality per $10^5$ births. Significance (two-tailed) compared with CM for all DDs: *0.01 < $P$ < 0.05, **0.001 < $P$ < 0.01, ***$P$ < 0.001.

© Cancer Research Campaign 1998

**Table 4** Variation between DDs in cumulative childhood cancer mortality (deaths aged 0–15 years in the period 1953–80). Multivariate analysis (Poisson regression)

| Parameter (units) | Rate ratio (95% confidence interval) All cancers | Leukaemias and lymphomas | Solid cancers |
|---|---|---|---|
| Socioeconomic family | | | |
| 1 Suburban and growth areas | 1.00 | 1.00 | 1.00 |
| 2 Rural and resort areas | 0.95 (0.88–1.03) | 1.00 (0.90–1.12) | 0.90 (0.80–1.00) |
| 3 Traditional industry and mining areas | 0.87 (0.81–0.92) | 0.90 (0.82–0.98) | 0.83 (0.76–0.91) |
| 4 Service centres | 0.82 (0.76–0.87) | 0.81 (0.74–0.88) | 0.82 (0.75–0.90) |
| 5 Areas with much LA housing | 0.79 (0.72–0.86) | 0.80 (0.70–0.91) | 0.77 (0.67–0.88) |
| 6 Inner London | 0.80 (0.67–0.96) | 0.89 (0.70–1.13) | 0.72 (0.55–0.94) |
| Birth density (births $ha^{-1}$ $year^{-1}$) | | | |
| Lowest quartile (0–0.01292) | 1.00 | 1.00 | 1.00 |
| Second quartile (0.01293–0.0528) | 1.07 (0.97–1.19) | 1.10 (0.95–1.26) | 1.04 (0.90–1.21) |
| Third quartile (0.0529–0.1882) | 1.21 (1.09–1.34) | 1.24 (1.07–1.43) | 1.18 (1.01–1.37) |
| Highest quartile (0.1883–1.4590) | 1.23 (1.11–1.36) | 1.25 (1.09–1.44) | 1.21 (1.04–1.40) |
| Birth year (1953–64) | 0.99 (0.98–1.00) | 0.98 (0.97–0.99) | 1.00 (1.00–1.01) |
| Radon (Bq $m^{-3}$) | 1.07[a] (1.02–1.12) | 1.06[a] (0.99–1.12) | 1.08[a] (1.02–1.15) |

[a]Rate ratio shows cumulative mortality for twice the mean value compared with the CM for the mean value (27.01 Bq $m^{-3}$).

Several additional variables were incorporated into the regression model and tested, but this did not improve the predictions or significantly reduce the scaled deviance. These additional tested variables included the socioeconomic clusters and measured indoor and outdoor gamma radiation levels. However, the optimal model (Table 4) left large residuals (i.e. large differences between observed and predicted values for some DDs), indicating the presence of important unidentified influences that were not reflected in the variables available for analysis.

### DISCUSSION

These analyses demonstrate the presence of significant heterogeneities in the geographical distribution of childhood cancer mortality in the UK according to place of birth, on a scale corresponding with the 10-km squares of the National Grid (i.e. demographic districts). There was no evidence from the Smans statistic of larger scale aggregations of high-mortality DDs and the pattern for both of the main classes of childhood cancer appeared as a scatter of higher risk areas separated by others of lower or average risk. This focused attention firmly upon variations between individual DDs and upon intrinsic local features resolvable to a scale of 10 km or less. Univariate analyses revealed significant differences in mortality between DDs with different socioeconomic characteristics, and multivariate Poisson regression showed that some of the birth place heterogeneity could be explained by the independent effects of variations in birth density, in the demographic and socioeconomic characteristics of the different districts, and by indoor radon exposure.

However, our analyses had certain limitations. One problem was the difference in time frame between the socioeconomic classification used (based on the 1971 census) and the study period (births 1953–64). Our aim was to describe the nature of the geographical distribution of childhood cancers in more meaningful and detailed terms than in our previous studies, when we reported crude gradients related to the easting of the place of birth (Knox et al, 1988). We wanted to characterize areas with high or low rates and, for the period for which we had complete data (1953–64 birth cohorts), the classification based on the 1971 census variable was the only appropriate tool that we could find. Changes in the character of some areas between the period 1953–64 and 1971 may have occurred but should have the effect of making it more difficult to demonstrate associations between area types and childhood cancer rates. As it is, we were able to confirm previously reported associations of high childhood cancer risks with births in areas classed as New Towns, 'rural growth' and 'rapid growth' areas.

A second problem is that the explanatory power of ecological regression is totally dependent upon the inclusion of aetiologically relevant factors in the model and relies on the assumption that the population characteristics used to describe an area accurately reflect the characteristics of the affected individuals in that population. Although the Poisson models indicated significant associations of CM with socioeconomic factors and exposure to indoor radon, none of the available variables alone, nor all of them together, successfully explained the whole of the heterogeneity between the DDs. This implies the presence of additional risk factors within individual DDs that were unrelated or only weakly related to the variables to which we had access. Some of these factors may be separately located in yet smaller areas, and this could explain some of the apparent inconsistencies in our findings. For example, we found that childhood cancer risk increased with relative affluence, as well as being high in suburban environments and in New Towns, yet it was also greater in areas of high population density (which are usually areas of low socioeconomic status); and, despite the latter, was lower than average in inner London and in Glasgow. The findings of the Poisson regression indicate a particularly powerful association of CM with zones whose population densities are greater than the median, yet also indicate increased risk with increasing socioeconomic status of areas within the separate density bands. Other workers have also reported somewhat confusing results: Draper et al (1991) found higher rates in rural (low population density) compared with urban (high population density) areas, and Langford and Bentham (1993) found a significant deficit of childhood acute lymphoblastic leukaemia in large service centres and cities, with presumably high population densities. Muirhead (1995) in an

 © Cancer Research Campaign 1998

investigation of census tracts in three metropolitan regions in the USA found a significant trend of increasing leukaemia risk with increasing population density, but found no relationship with level of income or education. These complex findings invite consideration of hazards that might be distributed in a comparably complex fashion. Three main aetiological hypotheses deserve special consideration. They are (a) radiation exposure, (b) exposure to chemical environmental hazards and (c) exposure to infection.

### Radiation exposure

The significant relationship with indoor levels of radon is consistent with an earlier finding derived from these same data. We demonstrated (Knox et al, 1988) a significant effect of outdoor gamma radiation and birth place (easting), but the analysis did not have information on radon exposure, nor did it include a measure of socioeconomic classification of area of child's birth, having instead a simpler variable measuring urban/rural status. From the Poisson modelling reported here, even though indoor gamma did improve the fit in the absence of radon, once radon is in the model addition of indoor or outdoor gamma does not significantly decrease the scaled deviance. Thus radon appears to be the more important radiation variable. Similarly easting and/or northing become redundant if socioeconomic family is in the model, implying that easting was acting as a proxy measure of socioeconomic classification of area.

The radon effect was of similar size in each of the diagnostic groups, but just failed to reach statistical significance among the leukaemias and lymphomas. The size of the radon effect was such that, compared with the median radon level of 21 Bq.m$^{-3}$, in areas with radon levels of 63 Bq m$^{-3}$ (5% of DDs had radon levels at or above this value) the rate ratio for all childhood cancers was 1.11 (95% confidence interval (CI) 1.04–1.19). These results indicate that in such areas around 10% (95% CI 3–16%) of childhood cancers may be attributable to the high radon levels. This estimate is compatible with that of Henshaw et al (1990) who estimated that up to 15% of childhood cancers could be attributed to an average radon exposure of 50 Bq m$^{-3}$. At these levels of exposure, the proportion of cases likely to be due to radon exposure is similar to that associated with childhood cancer risk after irradiation of the fetus in utero by radiography of the abdomen of the pregnant mother (Stewart et al, 1958; Bithell and Stewart, 1975; Muirhead and Kneale, 1989).

These radon results contrast with the findings of Muirhead et al (1991) and Richardson et al (1995) who could detect no consistent, significant effect of indoor radon on childhood cancer incidence. However, both analyses were based on location at time of diagnosis and did not use a birth-cohort approach. The indoor radon measurements used in the analyses reported here were made by the NRPB in the 1980s, many years after the study period (1953–64 births), but no earlier national data exist with which to investigate covariation of childhood cancer rates with radon levels. It is possible that in the intervening years there may have been changes in the housing stock (e.g. installation of double glazing, improvements in insulation) that may have altered radon exposure levels. Such changes may not have happened uniformly across the whole country but, for non-geographically uniform changes in housing to account for the association between childhood cancer rates and radon levels, these housing stock changes would themselves have to be associated with some other risk factor for childhood cancer; a reasonable candidate would seem to be the socioeconomic classification variable used in our analyses. In fact, there is a significant negative correlation between radon and socioeconomic family, and it may be that, although both radon and socioeconomic family exert independent effects in the regression model, some of the radon effect might be a reflection of socioeconomic factors not fully accounted for in the socioeconomic classification variable used in our analyses.

### Exposure to chemical environmental hazards

It was interesting that, at this geographical scale of analysis, there was no evidence of an increase in mortality from childhood cancers in industrialized areas, which might have been expected if exposure to the effects of toxic pollution was involved in their aetiology. However, using analyses conducted at a finer geographical level, we have found significant short range excesses of childhood cancers associated with certain types of industrial installations (Knox and Gilman, 1997). The apparent inconsistency of our present findings is possibly because of the inability of analysis at the larger scale of 10-km squares to detect the short-range effects of local discrete hazards.

### Exposure to infection

Many different infective mechanisms have been proposed, particularly with respect to the leukaemias. They have been tentatively invoked to explain notable 'epidemics' (Heath and Hasterlik, 1963), as well as more widespread, small-scale transient clusters (Smith, 1982; Draper, 1991). Such studies have related mainly to times and places of onsets and, if infection is to blame, it may be in relation to the promoting and precipitating phases of the disease process rather than to its initiation. However, these localized time–space concentrations could not easily explain geographical concentrations accumulated over many years, as demonstrated here. Other postulated infectious mechanisms, more capable in this last respect, have included mother–child virus transmissions with subsequent immune tolerance, and double infections when very early exposure results in cell transformation while reinfection results in clone proliferation (Alexander, 1993). Both such processes might be more frequent in population-dense areas.

A more complex epidemiological hypothesis, proposed in recent years (Kinlen, 1988; Kinlen et al, 1990, 1993), depends upon population-mixing in developing areas, where the immigrants' previous exposures to an immunizing infection differ from those of the longer term residents. A family of specific submodels can then be generated, depending on whether the risk of leukaemia is supposed to depend specifically upon age at infection (Greaves, 1988), whether immunity is supposed to be always permanent or sometimes transient, whether the immune immigrants enhance herd immunity and so reduce normal transmission among the earlier residents, whether a minority chronic carrier state is envisaged, and so on.

We found that birth areas with the highest subsequent childhood cancer mortalities included those classed as suburban and growth areas (areas of rural growth for the leukaemias and lymphomas; areas of rapid growth for the solid cancers) and New Towns (for the leukaemias and lymphomas). These groups are characterized by influxes of people from a wide range of areas to previously isolated locations, with a consequent mixing of populations from different backgrounds. In common with Kinlen et al (1990), we did not find elevated rates in overspill areas, i.e. areas where the

© Cancer Research Campaign 1998

incoming population came from a nearby, well-mixed large conurbation or other tightly defined geographical area, probably with similar infectious exposure backgrounds and containing few susceptibles. Stiller and Boyle (1996) also found that the effect of population-mixing on childhood leukaemia risk increased with the increasing diversity of areas from which the incoming population was derived.

The high observed mortalities among children born in growth areas and New Towns fit well with a general population-mixing model provided that the effect somehow carries over to generations born in these areas after the first mixing has taken place. It fits less well with the association of excess risk in population-dense and presumably high-transmission areas, from which the immigrants may have come. It has been suggested that ready transmission of infection prevents the accumulation of susceptibles (Langford and Bentham, 1993) and, while this may explain the apparent deficits of childhood cancer mortality in inner London or Glasgow specifically, it is scarcely compatible with our finding of a wider association of increased risk with increased population density. One problem is that available information on the birth density and the socioeconomic characteristics of the area of birth do not allow accurate quantification of migration levels or population-mixing or infectious transmission. Only tentative comments on a population-mixing infective hypothesis of the origins of childhood leukaemias (or other childhood cancers) are possible. But at least one positive conclusion is justified. If these correlations do indeed represent an infective mechanism, then the marks of its social mediation are as powerful for the solid cancers as for the leukaemias and lymphomas, and the outcomes are not limited to malignancies of the immune system alone.

It must be emphasized again that none of the models gave a sufficient explanation of the large-scale geographical variation in childhood cancer mortality. Our own parallel investigations of these data, and of a set of leukaemia and lymphoma registrations, indicate that there are other important influences affecting childhood cancer risk, and that they themselves must be distributed unevenly and probably on a finer scale than the heterogeneities examined above (Gilman, 1992; Knox, 1994; Gilman and Knox, 1995). The investigation of these influences calls for a different form of analysis on a much smaller scale, and it is the subject of ongoing work (Knox and Gilman, 1996, 1997).

### ACKNOWLEDGEMENTS

We gratefully acknowledge the help received from Professor AM Stewart and Mr GW Kneale in using the data held by the Oxford Survey of Childhood Cancers. This study was carried out as part of a research programme funded by the Medical Research Council and subsequently by the Three Mile Island Public Health Fund (USA).

### REFERENCES

Alexander FE (1993) Viruses, clusters and clustering of childhood leukaemia: a new perspective? *Eur J Cancer* **29A**: 1424–1443

Baron JA (1984) Cancer mortality in small areas around nuclear facilities in England and Wales. *Br J Cancer* **50**: 815–824

Beral V, Roman E and Bobrow M (eds) (1993) *Childhood Cancer and Nuclear Installations*. BMJ Publishing Group: London

Bithell JF and Stewart AM (1975) Pre-natal irradiation and childhood malignancy: a review of British data from the Oxford Survey. *Br J Cancer* **31**: 271–287

Bithell JF, Dutton SJ, Draper GJ and Neary NM (1994) Distribution of childhood leukaemias and non-Hodgkins lymphomas near nuclear installations in England and Wales. *Br Med J* **309**: 501–505

Clayton D and Bernardinelli L (1992) Bayesian methods for mapping disease risk. In *Geographical and Environmental Epidemiology. Methods for Small-Area Studies*, Elliott P, Cuzick J, English D and Stern R. (eds), pp.205–220. WHO, University Press: Oxford

Cook-Mozaffari PJ, Darby SC, Doll R, Forman D, Hermon C, Pike MC and Vincent TJ (1989) Geographical variation in mortality from leukaemia and other cancers in England and Wales in relation to proximity to nuclear installations 1969–1978. *Br J Cancer* **59**: 476–485

Craig J (1977) *Grid References of Centres of Population Great Britain 1971*. OPCS Occasional Paper 1

Darby SC and Doll R (1987) Fallout, radiation doses near Dounreay, and childhood leukaemia. *Br Med J* **294**: 603–607

Draper GJ (ed.) (1991) *The Geographical Epidemiology of Childhood Leukaemia and Non-Hodgkin Lymphomas in Great Britain, 1966–83*. Studies on Medical and Population Subjects No.53. HMSO: London

Draper GJ, Vincent TJ, O'Connor CM and Stiller CA (1991) Socioeconomic factors and variations in incidence rates between County Districts. In *The Geographical Epidemiology of Childhood Leukaemia and Non-Hodgkin Lymphomas in Great Britain, 1966–1983*, Draper GJ. (ed.), pp.37–45. Studies on Medical and Population Subjects No. 53. HMSO: London

Forman D, Cook-Mozaffari PJ, Darby S, Davey G, Stratton I, Doll R and Pike M (1987) Cancer near nuclear installations. *Nature* **329**: 499–505

Gilman EA (1992) *The Spatial and Temporal Distribution of Childhood Cancers in Britain*. PhD thesis, University of Birmingham

Gilman EA and Knox EG (1995) Childhood cancers: space-time distribution in Britain. *J Epidemiol Comm Hlth* **48**: 158–163

Greaves MF (1988) Speculations on the cause of childhood acute lymphoblastic leukaemia. *Leukaemia* **2**: 120–125

Green BMR, Lomas PR, Bradley EJ and Wrixon D (1989) *Gamma Radiation Levels Outdoors in Great Britain* (NRPB-R191). HMSO: London

Heath CW and Hasterlik RJ (1963) Leukemia among children in a suburban community. *Am J Med* **34**: 796–812

Henshaw DL, Eatough JP and Richardson RB (1990) Radon as a causative factor in induction of myeloid leukaemia and other cancers. *Lancet* **335**: 1008–1012

Kinlen L (1988) Evidence for an infective cause of childhood leukaemia: comparison of a Scottish new town with nuclear reprocessing sites in Britain. *Lancet* **2**: 1323–1327

Kinlen LJ, Clarke K and Hudson C (1990) Evidence from population mixing in British New Towns 1946–85 of an infective basis for childhood leukaemia. *Lancet* **336**: 577–582

Kinlen LJ, O'Brien F, Clarke K, Balkwill A and Matthews F (1993) Rural population mixing and childhood leukaemia: effects of the North Sea oil industry in Scotland, including the area near Dounreay nuclear site. *Br Med J* **306**: 743–748.

Knox G (1964) Epidemiology of childhood leukaemia in Northumberland and Durham. *Br J Prev Soc Med* **18**: 17–24

Knox EG (1994) Leukaemia clusters in childhood: geographical analysis in Britain. *J Epidemiol Comm Hlth* **48**: 369–376

Knox EG and Gilman EA (1996) Spatial clustering of childhood cancers in Great Britain. *J Epidemiol Comm Hlth* **50**: 313–319

Knox EG and Gilman EA (1997) Hazard proximities of childhood cancers in Great Britain from 1953–80. *J Epidemiol Comm Hlth* **51**: 151–159

Knox EG, Stewart AM, Kneale GW and Gilman EA (1987) Prenatal irradiation and childhood cancer. *J Radiol Prot* **7**: 177–189

Knox EG, Stewart AM, Gilman EA and Kneale GW (1988) Background radiation and childhood cancers. *J Radiol Prot* **8**: 9–18

Langford IH and Bentham G (1993) Epidemiology of childhood leukaemia. *Br Med J* **307**: 445–446

McCullagh P and Nelder JA (1983) *Generalised Linear Models*. Chapman and Hall: London

Muirhead CR (1995) Childhood leukemia in metropolitan regions in the United States: a possible relation to population density? *Cancer Causes Control* **6**: 383–388

Muirhead CR and Kneale GW (1989) Prenatal irradiation and childhood cancer. *J Radiol Prot* **9**: 209–212

Muirhead CR, Butland BK, Green BMR and Draper GJ (1991) Childhood leukaemia and natural radiation. *Lancet* **1**: 503–504

OPCS (1953–64) *Vital Statistics for Local Authorities*. Volumes for the years 1953 to 1964. HMSO: London

OPCS (1975) *Reorganisation of Local Government Areas. Correlation of New and Old Areas*. HMSO: London

Registrar General for Scotland (1953–64) Annual Reports for the years 1953 to 1964. General Register Office: Scotland

© Cancer Research Campaign 1998

Richardson S, Monfort C, Green M, Draper G and Muirhead C (1995) Spatial variation of natural radiation and childhood leukaemia incidence in Great Britain. *Stat Med* **14**: 2487–2501.

Roman E, Beral V, Carpenter L, Watson A, Barton C, Ryder H and Aston DL (1987) Childhood leukaemia in the West Berkshire and Basingstoke and North Hampshire District Health Authorities in relation to nuclear establishments in the vicinity. *Br Med J* **294**: 597–602

Smans M (1989) Analysis of spatial aggregation. In *Cancer Mapping*, Boyle P, Muir CS and Grundmann E. (eds), pp.83–86. Springer: Berlin

Smith PG (1982) Spatial and Temporal Clustering. In *Cancer Epidemiology and Prevention*, Schottenfeld D and Fraumeni JF. (eds), pp. 391–407 Saunders: Philadelphia

Stewart AM, Webb J and Hewitt D (1958) A survey of childhood malignancies. *Br Med J* **1**: 1495–1508

Stiller CA and Boyle PJ (1996) Effect of population mixing and socioeconomic status in England and Wales, 1979–85, on lymphoblastic leukaemia in children. *Br Med J* **313**: 1297–1300

Stiller CA and Draper GJ (1982) Trends in childhood leukaemia in Britain 1968–1978. *Br J Cancer* **45**: 543–551

Stiller CA, O'Connor CM, Vincent TJ and Draper GJ (1991) The National Registry of Childhood Tumours and the leukaemia/lymphoma data for 1966–1983. In *The Geographical Epidemiology of Childhood Leukaemia and Non-Hodgkin Lymphomas in Great Britain, 1966–1983.* Draper GJ. (ed.), pp.7–16. Studies on Medical and Population Subjects No. 53. HMSO: London

Vianna NJ, Greenwald P, Brady J and Polan AK (1972) Hodgkins disease: cases with features of a community outbreak *Ann. Intern Med* **77**: 169–180

Webber R and Craig J (1978) *Socio-economic Classification of Local Authority Areas*. Studies on Medical and Population Subjects No. 35. HMSO: London

Wrixon AD, Green BMR, Lomas PR, Miles JCH, Cliff KD, Francis EA, Driscoll CMH, James AC and O'Riordan MC (1988) *Natural Radiation Exposure in UK Dwellings* (NRPB-R190). HMSO: London

© Cancer Research Campaign 1998